# Three-Dimensional Bone Substitutes for Oral and Maxillofacial Surgery: Biological and Structural Characterization

**DOI:** 10.3390/jfb9040062

**Published:** 2018-11-08

**Authors:** Gianluca Turco, Davide Porrelli, Eleonora Marsich, Federica Vecchies, Teresa Lombardi, Claudio Stacchi, Roberto Di Lenarda

**Affiliations:** 1 Department of Medical Sciences, University of Trieste, Piazza dell’Ospitale 1, I-34125 Trieste, Italy; dporrelli@units.it (D.P.); emarsich@units.it (E.M.); federica.vecchies@gmail.com (F.V.); claudio@stacchi.it (C.S.); rdilenarda@units.it (R.D.L.); 2 Private Practice, Studio Odontoiatrico Hesire, I-87011 Cassano allo Ionio, Italy; drteresalombardi@libero.it

**Keywords:** biomaterials, dental materials, tissue engineering, scaffolds, bone graft materials, cell colonization, porosity, regenerative medicine, micro-computed tomography

## Abstract

Background: Bone substitutes, either from human (autografts and allografts) or animal (xenografts) sources, suffer from inherent drawbacks including limited availability or potential infectivity to name a few. In the last decade, synthetic biomaterials have emerged as a valid alternative for biomedical applications in the field of orthopedic and maxillofacial surgery. In particular, phosphate-based bone substitution materials have exhibited a high biocompatibility due to their chemical similitude with natural hydroxyapatite. Besides the nature of the biomaterial, its porous and interconnected architecture is essential for a correct osseointegration. This performance could be predicted with an extensive characterization of the biomaterial in vitro. Methods: In this study, we compared the biological, chemical, and structural features of four different commercially available bone substitutes derived from an animal or a synthetic source. To this end, µ-CT and SEM were used to describe the biomaterials structure. Both FTIR and EDS analyses were carried out to provide a chemical characterization. The results obtained by these techniques were correlated with cell adhesion and proliferation of the osteosarcoma MG-63 human cell line cultured in vitro. Results: The findings reported in this paper indicate a significant influence of both the nature and the structure of the biomaterials in cell adhesion and proliferation, which ultimately could affect the clinical performance of the biomaterials. Conclusions: The four commercially available bone substitutes investigated in this work significantly differed in terms of structural features, which ultimately influenced in vitro cell proliferation and may so affect the clinical performance of the biomaterials.

## 1. Introduction

Bone regenerative medicine aims to repair bone defects generated by congenital abnormalities, trauma or disease. Graft materials are often used in both maxillofacial and orthopedic procedures to regenerate lost tissue. Autogenous bone grafts (i.e., transplanted within the same individual) have been and are still considered the most effective substitution material due to their low antigenicity and high reliability [1,2,3,4]. Despite these advantages, the use of autogenous grafts faces inherent limitations such as limited bone source; anatomical, structural, and surgical limitations; post-operative pain; high resorption rates during healing; and donor site morbidity [5,6,7]. To overcome these drawbacks, allografts (obtained from different individuals of the same species) and xenografts (obtained from different species (e.g., bovine, porcine and equine) are considered as the most common alternatives to autografts [8,9,10,11,12]. However, these two bone substitutes are not exempt of shortcomings. On the one hand, allografts are limited by limited volumetric stability and potential infectivity. On the other hand, xenografts present some disadvantages associated with the immune response and potential contamination by prions [4,5,13,14,15]. Over the last decade, synthetic grafts, also known as scaffolds or alloplasts, have been developed in order to promote bone growth without any risk of disease transmission and no need for second surgical sites. These biomaterials are becoming increasingly used as bone substitutes for medical purposes. Ideally, they are conceived to mimic native bone architectures and to act as temporary three-dimensional osteoconductive supports for cell colonization and mineral matrix deposition [16,17]. Several organic, natural or synthetic biomaterials have been developed for various applications in periodontology, oral surgery, and orthopedics (sinus floor elevation, periodontal infrabony defects, alveolar ridge augmentation, spine fusion, maxillofacial reconstruction, and ear implants to name a few) [4,13,18,19]. In addition to their osteoconductive properties, the last and more recent generation of biomaterials aims to prevent the insurgence of infections including antibacterial agents (e.g., antimicrobial peptides or silver nanoparticles, to cite some) in their matrix [20,21,22].

Each class of grafting materials used in oral and maxillofacial surgery (autologous bone, allogeneic bone, xenogeneic bone, and alloplasts) present different advantages and disadvantages for being evaluated in clinical practice (Table 1).

Synthetic calcium phosphate biomaterials have emerged as the most widely used biomaterials in medicine [5,19,23,24]. These biomaterials derive their great biocompatibility from their chemical similitude with the main inorganic component of natural human bones and teeth: hydroxyapatite (HAp) [25,26]. It is well known that HAp enhances cell differentiation, migration, and proliferation into the newly formed bone tissue [23,24,27]. Bone grafts based on synthetic HAp are nowadays available on the market in the form of cement, powders, granules or blocks [4,5]. Their regenerative performance depends on several mechanical, structural, morphological, chemical, and biological parameters [5]. Several efforts have been made to thoroughly characterize and describe bone substitutes involving a wide range of bioanalytical methods to predict the outcomes when implanted in vivo. To this end, several in vitro tests have been proposed in the literature to assess the scaffold biocompatibility and cell proliferation under controlled conditions [22,28,29]. Regarding the architecture of bone substitutes, it is known that a trabecular structure with open-cell pores greater than 200 µm in diameter and a high degree of interconnectivity and a porosity higher than 80% leads to an adequate biological integration within the host tissue [17,30]. On the other hand, the physical and chemical features of scaffolds are considered as paramount aspects to favor native cell colonization and proliferation [5,23].

In this context, the purpose of this study was to describe and compare the biological, chemical, and structural features of four different commercially available bone substitutes. In the present study, a natural bone mineral of bovine origin (Bio-Oss, Geistlich, Switzerland) and a synthetic porous HAp (Fisiograft Bone, Ghimas, Italy), both in the form of blocks and granules, were compared. The adhesion and proliferation of human osteoblast-like cells (bone osteosarcoma MG-63 cell line) were assessed by means of colorimetric cell metabolism assays and the results were correlated with the chemical and structural characterization of the materials. Given that the three-dimensional features are considered crucial, micro-computed tomography (µ-CT) and scanning electron microscopy (SEM) were used to assess the porosity and architecture of the samples. The null hypotheses formulated were that (i) the nature and (ii) the structure of the samples did not influence in vitro cell proliferation. The results indicate a clear influence of the three-dimensional features and of the nature of the material on cell proliferation.

## 2. Results

### 2.1. Cell Proliferation

**Figure 1:**
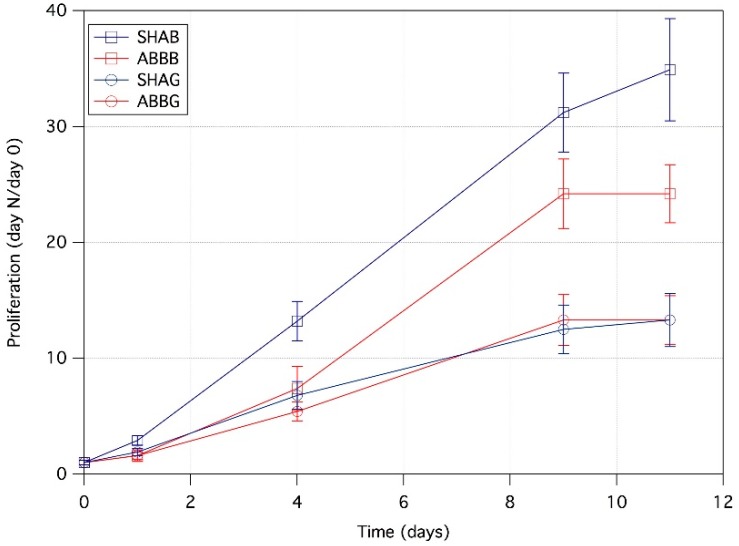
Cell proliferation of the osteosarcoma MG-63 human cell line as a function of time. The Alamar blue assay was performed at days 1, 4, 9, and 11 from initial seeding (day 0). Data (mean ± standard deviation) are expressed as the ratio between the fluorescence intensity at the specific time point and the fluorescence intensity of the same sample at day 0. SHAB = synthetic hydroxyapatite in blocks, ABBB = anorganic bovine bone in blocks, SHAG = synthetic hydroxyapatite in granules, ABBG = anorganic bovine bone in granules.

Figure 1 reports the cell proliferation results. Although a positive trend for proliferation was observed for all four groups, some differences were detectable. Cell proliferation on granules of both biomaterials was slower than that observed on the same products in block form. This can be deduced from the slope of the curves (i.e., the proliferation rate) and from the proliferation values at day 11, 34.9 ± 4.4 for SHAB, 24.2 ± 2.5 for ABBB, 13.3 ± 2.3 for SHAG, 13.3 ± 2.1 for ABBG (SHAB = synthetic hydroxyapatite in blocks, SHAG = synthetic hydroxyapatite in granules, ABBB = anorganic bovine bone in blocks, and ABBG = anorganic bovine bone in granules, see Materials and Methods Section). A comparison between the two products in the block form revealed that cells seeded on SHAB demonstrated a more rapid and constant proliferation from day one to day nine as compared to ABBB. At this time point, the cells seeded on ABBB, SHAG, and ABBG reached a plateau of proliferation (i.e., an interruption of cellular replication), whereas the cells seeded on SHAB continued to proliferate, albeit with an apparent slower rate, up to the last time point that was investigated (day 11).

### 2.2. Structural Analysis: µ-CT Results

**Figure 2:**
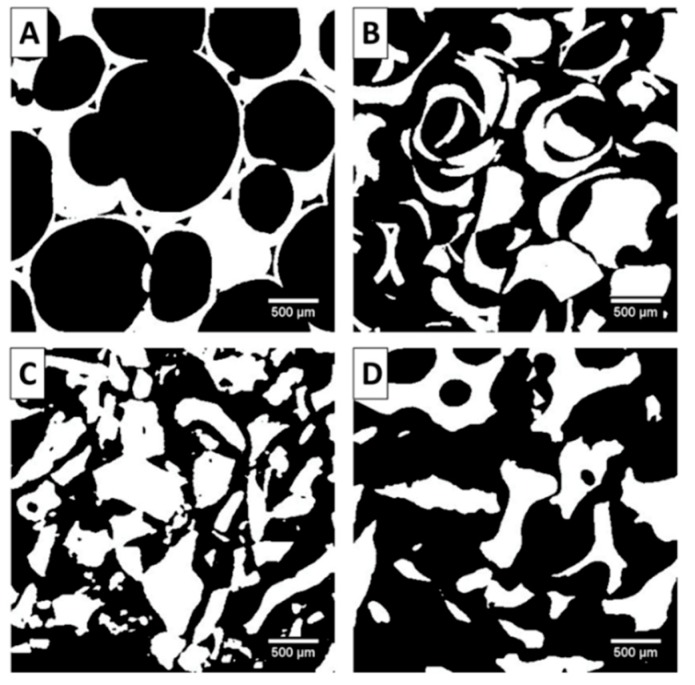
Segmented representation of a 3.2 × 3.2 mm^2^ (400^2^ pixels) slice of each considered µ-CT dataset. In these representations, the dark areas correspond to the empty spaces (voids), whereas white areas correspond to the solid matter. (**A**) SHAB = synthetic hydroxyapatite in blocks, (**B**) SHAG = synthetic hydroxyapatite in granules, (**C**) ABBB = anorganic bovine bone in blocks and (**D**) ABBG = anorganic bovine bone in granules. The scale bar represents 500 µm.

**Figure 3:**
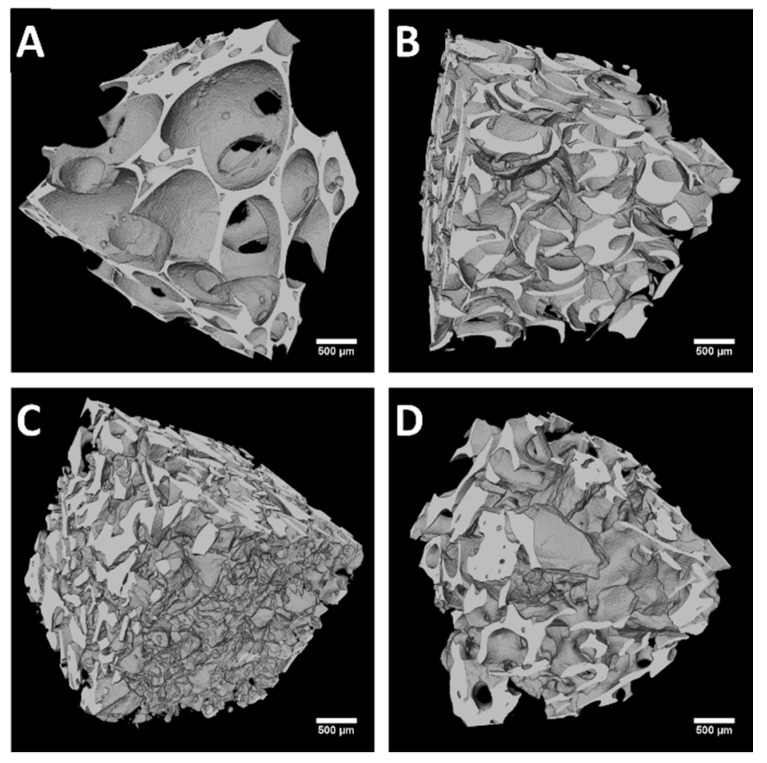
3D volume rendering of each µ-CT dataset. Each volume represented here has a 3.2 mm side length (400 pixels). (**A**) SHAB = synthetic hydroxyapatite in blocks, (**B**) SHAG = synthetic hydroxyapatite in granules, (**C**) ABBB = anorganic bovine bone in blocks, and (**D**) ABBG = anorganic bovine bone in granules. The scale bar represents 500 µm.

The results obtained from the µ-CT analyses are reported in Figure 2 and Figure 3 and Table 2. The cross sections (Figure 2) and the 3D volume rendering (Figure 3) of the investigated samples allowed for a first qualitative analysis of their architectural features. These images report a remarkably larger pore size (here defined as trabecular spacing) for the SHAB group as compared to the other groups. On the other hand, ABBB demonstrated a denser structure with the lowest trabecular spacing. Observing the granular form of the biomaterials that were investigated, SHAG appeared to have the highest packing (i.e., the way the granules were packed) of the particles as compared to ABBG. Another feature highly evident for SHAB was the interconnectivity of the pores. This is clearly observable in Figure 3A where the channels (throats) of interception between the pores are visible. This aspect was not present in ABBB where, as already mentioned, a more compact structure was observed. The interconnectivity of the granular material voids (interstices between the particles) depends on the particle size and shape. For SHAG and ABBG, the interstices appeared to have a random distribution (Figure 2B,D and Figure 3B,D). With the exception of interconnectivity, these structural features could be quantified by means of image analysis of the µ-CT slices (Table 2). In agreement with the qualitative analysis, SHAB presented the highest porosity (75%), followed by ABBG (65%). Both ABBB and SHAG exhibited the lowest porosity, 54% and 56%, respectively, with no statistically significant difference between these two values (Mann–Whitney *U*-test, *p* > 0.05). The trabecular thickness (Tb.Th) of the materials (i.e., the thickness of the walls between the pores or interstices) appeared to be statistically similar among SHAB, SHAG, and ABBG and was measured to be around 200 µm (Kruskal–Wallis, *p* > 0.05). On the contrary, ABBB showed the lowest Tb.Th (150 µm). The trabecular spacing (Tb.Sp), which could be considered as the average pore diameter, was found to be the highest for SHAB (992 µm) although SHAB also had the highest standard deviation (323 µm), which indicates a low level of homogeneity in the diameter of the pores. No statistically significant differences (Kruskal–Wallis, *p* > 0.05) were observed for the Tb.Sp values of the other three groups, ranging between 150 and 400 µm. The ratio between the bone surface (BS - the word “bone” conforms to literature usage; the word “solid” should actually be used instead) and the total volume (TV) was also evaluated (Table 2). For this parameter (BS/TV), the differences among the groups investigated were found to be statistically significant (Kruskal–Wallis, *p* < 0.05) with the highest value assessed for ABBB (9.0 mm^−1^), followed by SHAG (7.6 mm^−1^), ABBG (5.4 mm^−1^) and SHAB (3.4 mm^−1^). This trend was clearly in accordance with the porosity of the material.

### 2.3. Structural Analysis: SEM Results

**Figure 4:**
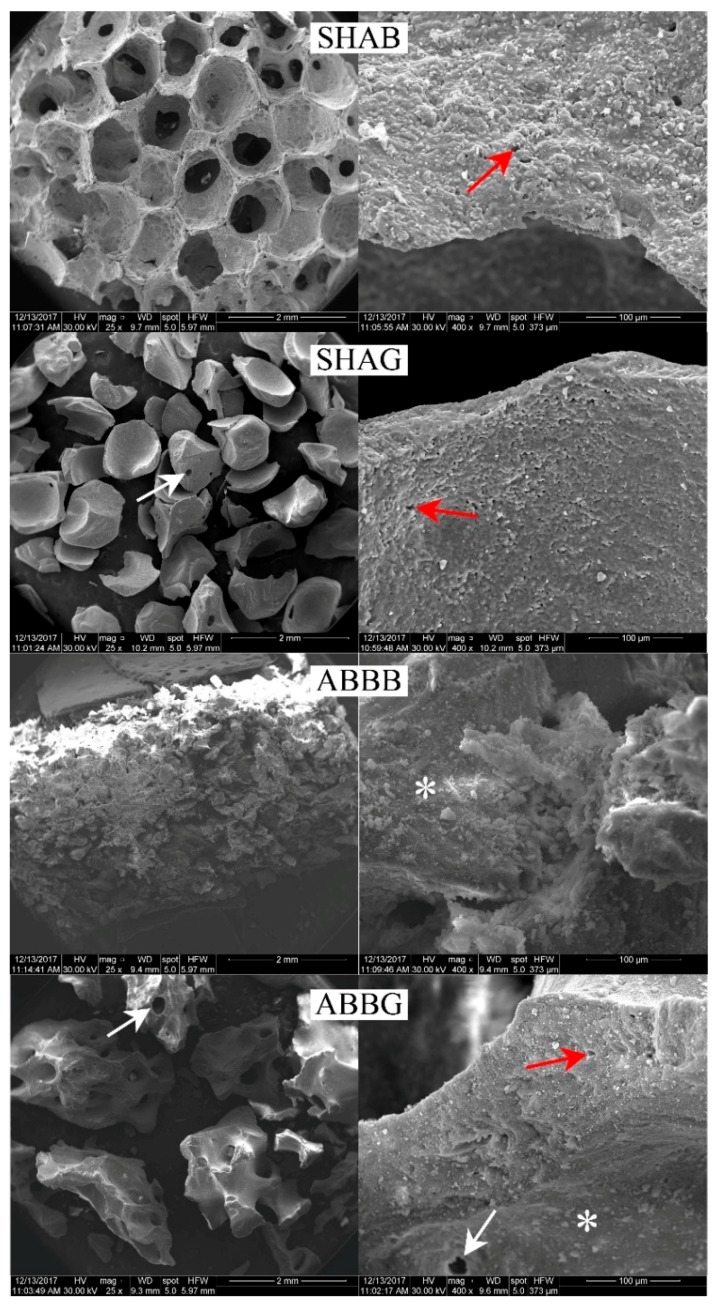
SEM micrographs, at two magnifications (25× and 400×), of four representative samples; one from each group. The white arrows indicate channels inside the granules, red arrows indicate micropores on the surface of the sample, white asterisks indicate the presence of debris on the surface of the sample. SHAB = synthetic hydroxyapatite in blocks, SHAG = synthetic hydroxyapatite in granules, ABBB = anorganic bovine bone in blocks, and ABBG = anorganic bovine bone in granules.

Figure 4 shows representative SEM micrographs illustrating the typical morphology of the four materials investigated here. The lowest magnification (25×) clearly shows the interconnectivity between the pores for the SHAB sample, the granular form of SHAG and SHAB and the compactness of ABBB. Moreover, at this magnification, the presence of channels inside the granules could be detected for both SHAG and ABBG (white arrows in Figure 4). At a higher magnification (400×), the surface roughness of samples could be observed. The SHAB and SHAG samples were highly similar and had micropores on the walls of the pores and granules respectively (red arrows in Figure 4). The presence of these micropores was also detected on the surface of ABBG and was not observed on ABBB. For these latter two groups, the presence of debris (white asterisks in Figure 4) could be seen at a high magnification (400×).

### 2.4. Fourier-Transformed Infrared Spectroscopy

**Figure 5:**
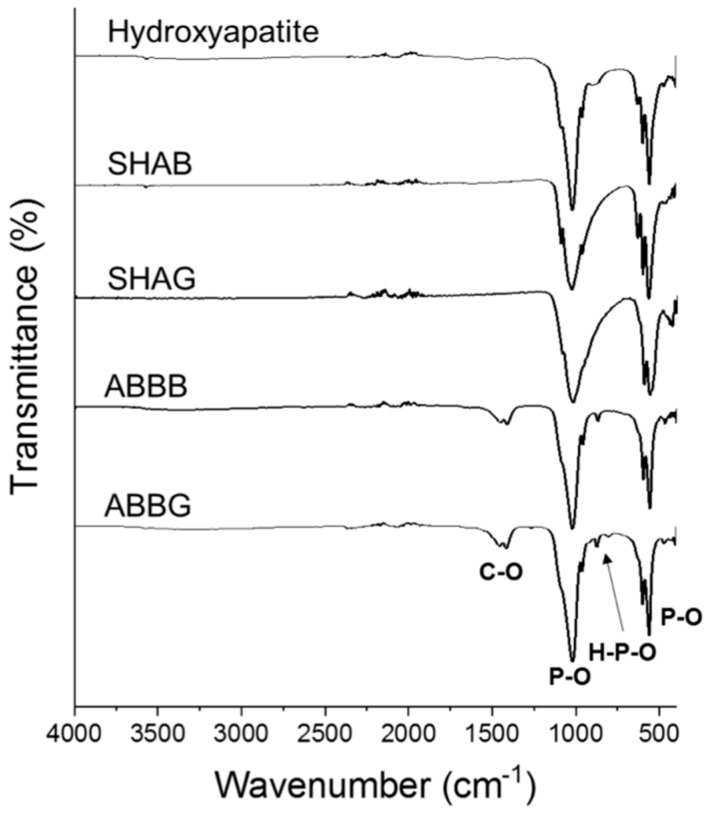
Fourier-transformed infrared spectra of the four biomaterials and a commercial hydroxyapatite powder (upper spectrum) with the bands assigned to the major chemical functional groups. SHAB = synthetic hydroxyapatite in blocks, SHAG = synthetic hydroxyapatite in granules, ABBB = anorganic bovine bone in blocks and ABBG = anorganic bovine bone in granules.

The infrared spectra are shown in Figure 5. This figure includes the spectra of the four biomaterials investigated here as well as the spectrum of a commercial HAp powder. HAp is the main constituent of the samples and therefore was used as a reference for this analysis. As expected, all of the four groups exhibited typical bands originating from this mineral. In particular, the phosphate stretching bands were detected at around 1010 cm^−1^ and 560 cm^−1^. Both forms of the anorganic bovine bone were present. In addition, a low-intensity double band at 1410–1460 cm^−1^ was observed that corresponds to the stretching vibration of the CO_3_^2−^ group. This band was absent in the spectra obtained for both the synthetic HAp and in the reference.

### 2.5. Energy Dispersive X-Ray Spectroscopy (EDS)

**Figure 6:**
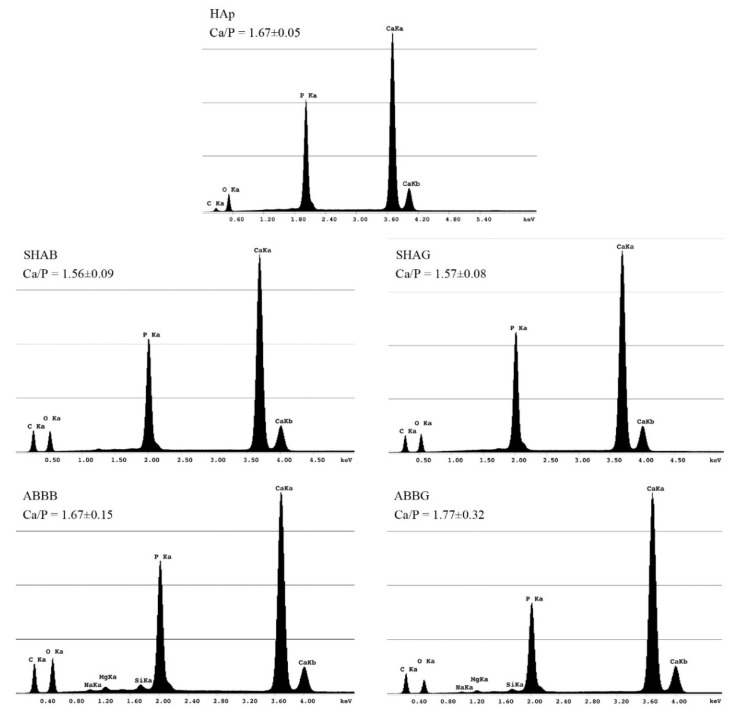
Energy dispersive X-ray spectroscopy (EDS) spectra for the four biomaterials and the commercial hydroxyapatite powder (upper spectrum). The peaks are labelled with the corresponding chemical element and electron shell. The characteristic peaks of calcium (Ca), phosphorous (P), oxygen (O) and carbon (C) can be observed in all of the samples. The presence of this latter element was ascribed to the carbon coating needed to properly scan the samples by means of SEM coupled with EDS. The ABBB and ABBG groups revealed the presence of other trace elements (less than 0.5% of the total) as silicon (Si), magnesium (Mg), and sodium (Na). For each spectrum, below the identification group acronym, the ratio between the detected amounts of calcium and phosphorous is reported (mean ± standard deviation, *n* = 4). SHAB = synthetic hydroxyapatite in blocks, SHAG = synthetic hydroxyapatite in granules, ABBB = anorganic bovine bone in blocks and ABBG = anorganic bovine bone in granules.

The EDS spectra are reported in Figure 6. It can be seen that all four of the materials investigated here exhibited the characteristic peaks for calcium (Ca), phosphorous (P), and oxygen (O), which are the main elemental species present in HAp: Ca_10_(PO_4_)_6_(OH)_2_. Carbon (C), related to the coating sputtered to ensure proper SEM and EDS observations, was also detected on all four of the groups and on the reference (commercial HAp powder). Moreover, for ABBB and ABBG, traces of silicon (Si), magnesium (Mg), and sodium (Na) were detected in amounts lower than 0.5% of the total elemental analysis. The stoichiometric Ca/P mole ratio of the pure HAp was calculated to be 1.67 from its chemical formula. This ratio (see text labels in Figure 6) was confirmed in all biomaterials involved in this study without any significant difference (Kruskal–Wallis test, *p* > 0.05).

## 3. Discussion

The main purpose of this study was the biological and structural characterization of four commercially available bone substitutes for regenerative applications. These biomaterials were chosen in the form of blocks or granules and from two different origins: xenograft and synthetic. From the data reported here, we observed a significant influence of both the nature and the structure of the biomaterials in cell adhesion and proliferation. These observations allowed us to reject both of the null hypotheses formulated in the introduction. The data on cell proliferation reported in Figure 1 highlights a clear dependence not only on the chemical composition but also on the morpho-structural features of the biomaterial itself. Characteristics such as the shape and size of trabecular materials have been already demonstrated to play an essential role in the materials’ performance [17,27,31,32,33,34,35,36]. In this work, using µ-CT image analysis and SEM, the authors assessed significant differences in the structure of the biomaterials (Figure 2, Figure 3 and Figure 4 and Table 2). From the quantitative characterization of the structure of each sample (Table 2), SHAB and ABBB, which demonstrated the best performance in terms of cell proliferation (Figure 1), appeared to have relevant differences. The porosity of SHAB was the highest (75%) and that of ABBB was the lowest (54%). On the contrary, the surface of the solid matter over the total volume (here defined as the ratio between the bone surface and total volume), appeared to have the lowest value for SHAB (3.4 mm^−1^) and the highest value for ABBB (9.0 mm^−1^). Therefore, potentially, ABBB had the highest surface area available for cell colonization. Cell proliferation assessed on this material showed a plateau at day 9 (Figure 1) indicating that the cells were no longer able to proliferate. This behavior is believed to reside in the compactness of this material, which, albeit exhibiting the highest BS/TV, showed the lowest average trabecular spacing (156 µm) and the lowest porosity (54%). This could possibly hamper cell spreading and scaffold colonization. In the literature, many authors seem to agree that interconnected porosity in the range of 70%–90% and an average pore size larger than 200 µm are essential for optimal cell growth and migration as well as for the diffusion of nutrients and catabolite exchange [17,30,35,36,37,38,39].

On the other hand, the scant performance of the granular materials in terms of cell proliferation was ascribed by the authors to both the packing characteristics of the particles and to their surface morphology. The SHAG samples, which are the same product as SHAB but in the granular form, exhibited a much lower porosity (56%) and a limited trabecular spacing (223 µm), which hindered cell proliferation from day 1. Moreover, the interconnectivity of the pores observed by µ-CT and SEM for the SHAB samples (Figure 3 and Figure 4) was completely absent on the same product in the granular form (SHAG). The granules of anorganic bovine bone (ABBG), albeit with almost optimal features in terms of structural characteristics, did not allow for efficient cell proliferation. The reason for this behavior is believed to reside in the surface properties of the particles. As highlighted by the SEM images, the surface of these particles was covered with debris (white asterisks in Figure 4). These debris are thought to limit the adhesion of cells on the biomaterial surface, therefore, obstructing cell proliferation. Even if debris were present on the ABBB surface as well, cells seeded on this biomaterial exhibited a higher proliferation rate as compared to ABBG. This difference could be correlated to the bone surface area available for cell proliferation, which was much lower for ABBG (5.4 mm^−1^) compared to ABBB (9.0 mm^−1^), confirming the paramount role of the structural features for cell behavior.

The presence of micropores on the surface of the sample (red arrows in Figure 4) has to be considered as a positive feature for in vivo applications. The literature is, indeed, accordant in reporting this aspect as beneficial for osseointegration, enhancing ionic exchange with body fluids and therefore improving cell adhesion, proliferation and differentiation [31,40,41,42,43]. Moreover, microporosity has been reported to generate capillary forces that draw fluids and cells on the surface of the scaffolds, enhancing the regenerative potential in vivo [30,44].

In the present work, the chemical characterization of the materials was performed by means of FTIR and EDS. Both of these techniques allowed for an exhaustive description of the chemical species detectable in the samples. As reported in Figure 5 and Figure 6, the samples investigated here and the chosen reference (HAp) exhibited similar behavior. As expected, the FTIR results (Figure 5) showed phosphate stretching bands for all the samples, indicating a high affinity with HAp. Moreover, the presence of the stretching vibration of the CO_3_^2−^ group in both forms of the anorganic bovine bone is in agreement with the observations made by Figueiredo and co-workers [31]. These authors correlate this band to the presence of carbonate apatite in the biomaterial due to its animal source. EDS analyses (Figure 6) indicate that the samples did not significantly differ in terms of elemental chemical composition and the Ca/P ratio. This latter feature is seen as a benefit for the biomaterials in terms of the similarity to the inorganic component of native bone tissue (i.e., HAp), which could enhance scaffold integration in vivo [45]. The presence of silicon in the anorganic bovine bone spectra has already been reported by Berberi and co-workers [46]. By means of X-Ray diffraction, these authors demonstrated that silicon is part of the calcium phosphate silicate hydroxide found in most commercial bone substitutes and in the autogenous bone itself. Therefore, its presence in the ABBB and ABBG samples was not surprising.

Besides the chemical similarity, the differences in the structural features observed here are expected to affect the performance of the materials investigated here once implanted in vivo.

## 4. Materials and Methods

### 4.1. Preparation of Biomaterials

The biomaterials selected for this study were synthetic HAp (Fisiograft Bone, Ghimas, Italy) and anorganic bovine bone (Bio-Oss, Geistlich, Switzerland). These materials were provided by the manufacturer in the form of blocks and granules. Hereafter, we refer to ABBB and ABBG for anorganic bovine bone in blocks and granules, respectively and to SHAB and SHAG for synthetic HAp in blocks and granules, respectively. Upon arrival, the materials were immediately divided into four groups: ABBB, ABBG, SHAB, and SHAG. Under sterile conditions, the blocks were cut into smaller cubes (5 mm lateral dimension, *n* = 8 for each manufacturer) and individually laid (one cube each well) in a non-treated 24-well plate for cell culture. This type of multi-well plate was chosen to avoid cell adhesion on the plastic and so to force cell interaction with the tested materials. In the same way, 125 µL of granules (*n* = 8 for each manufacturer) were added to the bottom of another identical 24-well plate (125 µL in each well). In the wells, the granules formed a compact and uniform layer with an average estimated thickness of 2 mm. This procedure allowed the authors to compare equal volumes of the biomaterials.

### 4.2. Cell Culture and Seeding

The osteosarcoma MG-63 (ATCC number: CRL-1427) human cell line was cultured in Dulbecco’s modified Eagle’s medium (DMEM) supplemented with 10% fetal bovine serum (FBS), 1% penicillin-streptomycin/1% l-glutamine at 37 °C and 5% pCO_2_. Prior to cell seeding, samples were conditioned by carefully pouring 1500 µL of complete DMEM on the samples and on an additional 4 samples for each group. These latter samples (blanks—scaffolds without cells) were used to define a background for cell proliferation (see next section). After 24 h, the excess medium was removed and a suspension of 5 × 10^3^ MG-63 cells in 50 μL of medium was gently loaded with a micropipette over the whole upper surface of the scaffolds with the exception of the blanks. After 4 h from cell seeding, 1500 µL of complete DMEM was added. Then, the two multi-wells were incubated in a humidified atmosphere of 5% CO_2_ at 37 °C.

### 4.3. Cells Proliferation and Viability

The growth rate of MG-63 cells was assessed as a function of time by using the Alamar blue assay according to the protocol provided by the manufacturer (Thermo Fisher Scientific, Life Technologies, Waltham, MA, USA). Assays (*n* = 8 for each group, *n* = 4 for blanks) were performed at 1, 4, 9, and 11 days from cell seeding. Briefly, at each time point, the samples were incubated with 200 µL of 10% Alamar blue in DMEM culture medium for 2 h in a dark and humidified atmosphere of 5% CO_2_ at 37 °C. The medium was then collected from each well and the fluorescence intensity was measured (λ_exc_ = 560 nm; λ_em_ = 590 nm) with a spectrofluorometer (GloMax Multi Detection System, Promega Corp., Madison, WI, USA). The background signal obtained from the blank scaffolds was subtracted from the sample values. For the sake of clarity, the authors would like to underline that these results are reported without a unit of measurement. This is due to the fact that proliferation was considered as the ratio between the fluorescence intensity at a specific time point and the fluorescence intensity of the same sample at day 0.

### 4.4. X-Ray Microcomputed Tomography

X-ray microcomputed tomography (µ-CT) of the samples was carried out using a custom-made cone-beam system called TomoLab [47]. The block samples were positioned directly onto the turntable of the instrument; the granulated samples were previously packed into 1.5 mL microcentrifuge test tubes. Data were collected using the following parameters: distance source-sample (F_OD_), 80 mm; distance source-detector (F_DD_), 250 mm; magnification, 3.1×; binning, 2 × 2; resolution, 8 µm; tomography dimensions (pixels), 2004 × 1335; slices dimensions (pixels), 1984 × 1984; number of tomographies, 1440; number of slices, 1332; E = 80 kV, I = 100 µA; exposure time, 5 s; filter = aluminium, 0.5 mm. The slice reconstruction process and the correction of beam hardening and ring artefacts were achieved using commercial software (Cobra EXXIM, Computing Corporation, Pleasanton, CA, USA). The input projections and output slices were represented by files (one file per projection and one file per slice) using arrays of 16-bit integers. The segmentation of the slices was performed by the Otsu’s method using Fiji open source processing software [48,49]. The BoneJ plugin, implemented in Fiji software, was used for the analysis of the samples after the segmentation process [50]. For each group, four cubic volumes of interest (VOI) with a side length of 3.2 mm were analyzed.

### 4.5. Scanning Electron Microscopy and Energy Dispersive X-Ray Spectroscopy

Topographical analysis of the surface of the sample was achieved using scanning electron microscopy (SEM). To this end, specimens were mounted on aluminum stubs covered with conductive double-sided carbon adhesive tape. Prior to SEM observation, samples were carbon coated (Sputter Coater K550X, Emitech, Quorum Technologies Ltd, Laughton, East Sussex, UK). Scanning electron microscopy (Quanta250 SEM, FEI, Hillsboro, OR, USA) was performed in secondary electron detection mode. The working distance was adjusted in order to obtain a suitable magnification; the accelerating voltage was set to 30 kV. The elemental composition of the scaffolds was determined using an Energy Dispersive X-Ray Spectroscopy (EDS) probe (Quanta250 FEI with EDAX probe, Hillsboro, OR, USA). The accelerating voltage varied between 10 and 30 kV; both spot-size and full frame acquisitions were performed by varying the scanning time from 5 to 15 min.

### 4.6. Fourier-Transformed Infrared Spectroscopy

Fourier-Transformed Infrared Spectroscopy (FTIR) was used to assess the chemical composition and the major functional groups present in the samples. Prior to analysis, samples from each of the four groups were homogenized using a mortar and pestle under dry conditions. Spectra were then recorded in the range 400–4000 cm^−1^ using a Nicolet 6700 spectrometer (Thermo Scientific, Milan, Italy) in attenuated total reflectance (ATR) mode. Each spectrum was collected at room temperature at a resolution of 6 cm^−1^ and the number of sample scans was 64. HAp powder was purchased from Sigma-Aldrich (St. Louis, MO, USA) and used as a reference.

### 4.7. Statistical Analysis

A statistical analysis was performed using SPSS Statistics 24 (IBM SPSS Statistics; SPSS Inc., Chicago, IL, USA). For the quantitative characterization of the structural features of the samples, a µ-CT dataset-based image analysis was carried out, after assessing the violation of the normality distribution (Kolmogorov–Smirnov test) and the homoscedasticity (Levine test) assumptions. Data sets were analyzed using non-parametric tests (Kruskal–Wallis test followed by, if significant, group comparisons with the Mann–Whitney *U*-test). Statistical significance was pre-set at α = 0.05.

## 5. Conclusions

The choice among different biomaterials and among different formulations of the same biomaterial represents an important step to optimize the clinical performance in oral and maxillofacial surgery, especially when dealing with sites with low regenerative potential (e.g., wide maxillary sinus cavities [51,52], vertical ridge augmentation procedures [53]). The four commercially available bone substitutes investigated in this work significantly differed in terms of structural features, which ultimately influenced in vitro cell proliferation. The findings of the present work highlight that the chemical composition of the biomaterial is not the sole parameter influencing the cellular response. Indeed, other factors, such as micro-architecture and porosity, must be taken into account to achieve better clinical results.

## Figures and Tables

**Table 1:** Advantages and disadvantages of the biomaterials used for bone regeneration.

Biomaterials	Advantages	Disadvantages
Autologous Bone	Osteoconduction; osteoinduction; no risks of cross infection or immune response; cost-effective	High morbidity; limited availability; limited dimensional stability over time
Allogeneic Bone	Osteoconduction; osteoinduction; low morbidity; unlimited availability	Theoretical risk of cross infection or immune response; limited dimensional stability over time; high costs
Xenogeneic Bone	Osteoconduction; low morbidity; unlimited availability; high dimensional stability over time	No osteoinductive properties; theoretical risk of cross infection or immune response; high costs
Alloplastic Materials	Osteoconduction; low morbidity; unlimited availability; variable dimensional stability over time; no risk of cross infection	No osteoinductive properties; theoretical risk of immune response; high costs

**Table 2:** Quantitative characterization of the structural features of the sample by means of a µ-CT dataset-based image analysis. Porosity is defined as the complementary measure of the ratio between the bone volume and total volume. The word “bone” conforms to literature usage; the word “solid” should actually be used instead. Tb.Th stands for trabecular thickness, whereas Tb.Sp stands for trabecular spacing. For the sake of readability, the ratio between the bone surface and total volume (BS/TV) is expressed as mm^−1^ instead of µm^−1^. Each value is expressed as the mean and the standard deviation of the parameters calculated for four cubic volumes of interest (VOI) with a side length of 3.2 mm. For the same parameter, groups identified with different lowercase letters are significantly different (Mann–Whitney *U*-Test, *p* < 0.05, lowercase letter a indicate the statistical group with the highest values, b mid-values, c and d lowest values). SHAB = synthetic hydroxyapatite in blocks, SHAG = synthetic hydroxyapatite in granules, ABBB = anorganic bovine bone in blocks and ABBG = anorganic bovine bone in granules.

Structural Parameters	SHAB	SHAG	ABBB	ABBG
Porosity (%)	75 ± 2 a	56 ± 2 c	54 ± 1 c	65 ± 1 b
Tb.Th (µm)	194 ± 100 a	207 ± 127 a	150 ± 59 b	193 ± 75 a
Tb.Sp (µm)	992 ± 323 a	223 ± 106 b	156 ± 71 b	397 ± 182 b
BS/TV (mm^−1^)	3.4 ± 0.4 d	7.6 ± 0.3 b	9.0 ± 0.1 a	5.4 ± 0.3 c

## Abbreviations

ABBB	anorganic bovine bone in blocks
ABBG	anorganic bovine bone in granules
EDS	energy dispersive X-ray spectroscopy
FTIR	Fourier transformed infrared spectroscopy
HAp	hydroxyapatite
µ-CT	micro-computed tomography
SEM	scanning electron microscopy
SHAB	synthetic hydroxyapatite in blocks
SHAG	synthetic hydroxyapatite in granules
VOI	volume of interest
